# Addressing child and adolescent obesity management in Ireland: identifying facilitators and barriers in clinical practice

**DOI:** 10.3389/fped.2023.1222604

**Published:** 2023-07-07

**Authors:** Farzana Ferdous, Niamh Arthurs, Louise Tully, Sarah O’Brien, Susan M. Smith, Aisling Walsh, Clodagh S. O’Gorman, Grace O’Malley

**Affiliations:** ^1^ Obesity Research and Care Group, School of Physiotherapy, RCSI University of Medicine and Health Sciences, Dublin, Ireland; ^2^ Health Service Executive (HSE) Health and Wellbeing Division, Dublin, Ireland; ^3^ Discipline of Public Health and Primary Care, Trinity College, Dublin, Ireland; ^4^ Department of Epidemiology, Division of Population Health Sciences, RCSI University of Medicine and Health Sciences, Dublin, Ireland; ^5^ School of Medicine, University of Limerick, Limerick, Ireland; ^6^ Department of Paediatrics, University Hospital Limerick, Limerick, Ireland

**Keywords:** adolescents, barriers, childhood obesity, healthcare professionals, overweight and obesity management

## Abstract

**Background:**

Ireland’s Model of Care for the Management of Overweight and Obesity outlines a plan for treating adolescent and child obesity (CO). However, engagement with key stakeholders is required to support its implementation and improve health services.

**Aim:**

This study aims to map the perceived barriers and facilitators related to CO management across healthcare settings, professional disciplines, and regions in the Republic of Ireland (ROI).

**Materials and methods:**

An online cross-sectional survey of registered healthcare professionals (HPs), designed to adhere to the Consolidated Framework for Implementation Research (CFIR), was co-developed by a project team consisting of researchers, healthcare professionals, and patient advocates. The survey was pilot tested with project stakeholders and distributed online to professional groups and via a social media campaign, between September 2021 and May 2022, using “SurveyMonkey.” Data were summarised using descriptive statistics and thematic analyses. Themes were mapped to the CFIR framework to identify the type of implementation gaps that exist for treating obesity within the current health and social care system.

**Results:**

A total of 184 HPs completed the survey including nurses (18%), physicians (14%), health and social care professionals (60%), and other HPs (8%). The majority were female (91%), among which 54% reported conducting growth monitoring with a third (32.6%) giving a diagnosis of paediatric/adolescent obesity as part of their clinical practice. Nearly half (49%) of the HPs reported having the resources needed for clinical assessment. However, 31.5% of the HPs reported having enough “time,” and almost 10% of the HPs reported having no/limited access to suitable anthropometric measurement tools. Most HPs did not conduct obesity-related clinical assessments beyond growth assessment, and 61% reported having no paediatric obesity training. CFIR mapping identified several facilitators and barriers including time for clinical encounters, suitable materials and equipment, adequate training, perceived professional competency and self-efficacy, human equality and child-centredness, relative priorities, local attitudes, referral protocols, and long waiting times.

**Conclusions:**

The findings provide actionable information to guide the implementation of the Model of Care for the Management of Overweight and Obesity in Ireland. Survey findings will now inform a qualitative study to explore implementation barriers and facilitators and prioritise actions to improve child and adolescent obesity management.

## Introduction

Obesity is a chronic disease and a significant risk factor for numerous additional non-communicable diseases (NCDs) (1). Recent data suggest that young people under 19 years of age are increasingly affected by obesity, which represents a significant global public health issue (2). According to the World Health Organization (WHO), nearly 400 million children and adolescents worldwide were estimated to be living with overweight or obesity in 2016 (3). Overweight and obesity affect nearly one in three children in the WHO European Region (4) and between 16% and 25% of children and adolescents in the Republic of Ireland (ROI) (5–7). Due to the impact of obesity on child health and development both in the short and long term, an Ireland National Health Service Executive (HSE) model of clinical care was developed to establish plans for the management of obesity in this population (8, 9). The model of care focuses on treatment and aims to complement the Obesity Policy and Action Plan, which incorporates obesity prevention (10). Healthcare in Ireland is state-funded for the most part, with 78% of health expenditure covered in 2021, and, in general, quality is good with mortality rates below the Organization for Economic Cooperation and Development (OECD) average (11). There are some concerns that a two-tier system is in place and waiting lists are unacceptably long for inpatient and outpatient services. Traditionally, the healthcare system was centred on hospital-based care, but recently broad reform has begun (termed Sláintecare) with the aim of increasing access to universal care and integrating primary, community, and hospital-based care (12). The emergence of the COVID-19 pandemic reinforced the importance of addressing obesity given that people living with obesity had a greater risk of severe disease following SARS-CoV-2 infection and considering that the implementation of COVID-19 restrictions was followed by a sharp rise in obesity among children (13–15). There is a pressing need to build capacity within health systems so that children and adolescents with obesity can access health services for assessment and appropriate treatment of obesity and related complications where present. In turn, it is important to understand how to best integrate obesity care for children and young people (CYP) in routine health services. Nevertheless, service users including healthcare professionals (HPs), parents, and young people are too frequently left out of, or insufficiently acknowledged in, health policies and plans, affecting their access to and the delivery of health and social care. Evidence suggests that many HPs are not adequately trained or otherwise equipped to deliver evidence-informed care (16–19). Optimal implementation of innovations and changes in practice for healthcare necessitates co-design and collaboration with frontline healthcare professionals who understand the contextual factors that might facilitate or hamper change in service design or delivery in their respective settings (20, 21). For example, the revised RE-AIM (reach, effectiveness, adoption, implementation, and maintenance) framework (22) describes the need to assess organisational and patient characteristics and perspectives to understand specific contexts. Limited data suggest that there is no consistent approach to assessment, diagnosis, management, or signposting for obesity in paediatric services in practice locally, but rather standalone interventions or partial interventions may be delivered in routine practice by individuals and services regionally (23–27). In Ireland, to realise the implementation of the HSE model of care for treating obesity, a fundamental understanding is required around (a) what HPs can currently deliver in terms of treatment and (b) what is needed to build capacity throughout the health system to improve identification, clinical assessment, treatment access, and treatment delivery and outcomes related to obesity in CYP.

This study aimed to identify the practices reported by HPs across health settings, professional disciplines, and geographical regions, in addressing obesity assessment and treatment in CYP in the Republic of Ireland. Secondary aims included assessing the views of participants about managing paediatric obesity in general and the level of relevant training among personnel.

## Methods and materials

### Study design

**Figure 1:**
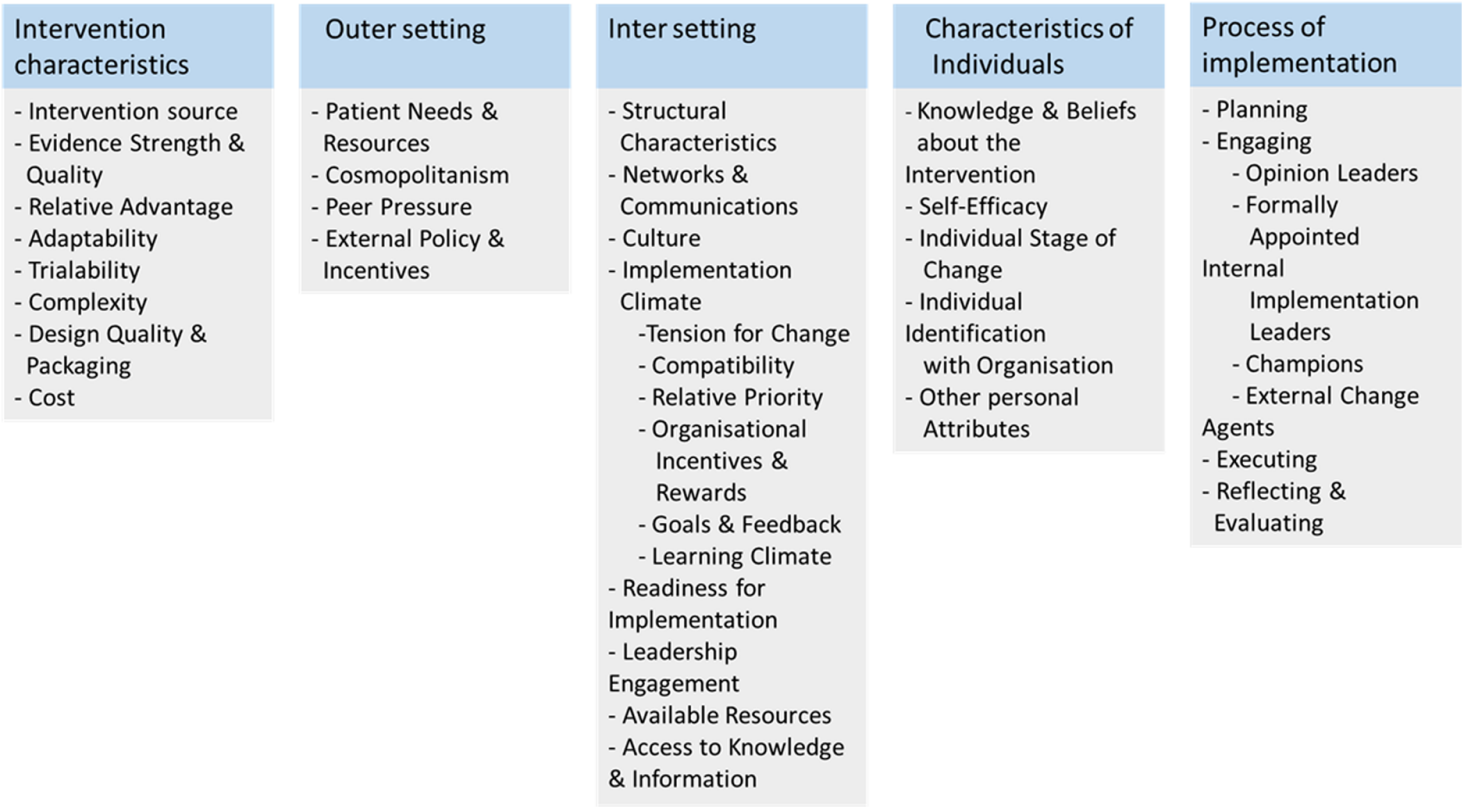
The Consolidated Framework for Implementation Research (CFIR) 2009 constructs.

The LANDSCAPE project aims to capture the views of stakeholders in the management of obesity in CYP, in order to inform the implementation of clinical services in Ireland, and is comprised of five distinct work packages (28). This paper reports HPs’ experiences in managing child and adolescent obesity in Ireland, which were collected via an anonymous online cross-sectional survey. The survey design was informed by a systematic review of clinical practice guidelines, which guided the definitions of obesity assessment and treatment used in the survey (29). It incorporated a semi-structured questionnaire, including the opportunity for free-text comments, was disseminated using the SurveyMonkey platform, and was designed in line with the CHERRIES checklist (30). The questionnaire contained 49 questions, which were developed using adapted items from previous studies in other settings (31). The Consolidated Framework for Implementation Research (CFIR) was used to categorise responses based on factors known to be important for implementing change or innovation to health services as described in Figure 1 (32). An iterative process of piloting and obtaining feedback from a multidisciplinary research team and patient representatives (adults/parents; *n* = 2) was followed. The survey is presented in Supplementary Appendix 1.

### Study population and sample size

All HPs, who are practising in primary-, secondary-, or tertiary-care public services, privately, or in third-sector organisations in the Republic of Ireland, were eligible to participate, if they delivered care to CYP, and were involved in care related to paediatric obesity management (e.g., dietitians, psychologists, physicians, physiotherapists, nurses, and social workers) (33).

Using the personnel census data from the public health system (Health Service Executive, HSE) and assuming a margin of error of ±5%, we estimated our target sample size to be *n* = 372. A sample size of *n* = 95 was estimated to be sufficient for a ±10% margin of error.

### Participant recruitment, eligibility criteria, and data collection

Participants self-screened for eligibility based on criteria listed on digital recruitment fliers and posts shared through purposive sampling on social media (Supplementary Appendix 2), in HP newsletters and through professional bodies, or at conferences/events. Snowball sampling was utilised through sharing of study information among professional and social media networks. Inclusion criteria were if the individual identified themselves as both:

• A HP working in health services in Ireland
• A HP working with children and adolescents

Following self-screening, participants were presented with a downloadable participant information sheet and contact information for queries and provided informed consent digitally prior to completing the survey. Responses were collected between September 2021 and May 2022. This study only includes data from those who completed the survey.

Participants provided demographic information (gender, discipline, role, setting, region, years of experience) and information related to study outcomes (Supplementary Appendix 1). Open-ended questions facilitated the collection of qualitative data throughout the questionnaire and to add context, where needed.

### Study variables

#### Outcome measures

The outcomes of interest for this included professionals’ current clinical practices, training received, self-efficacy, alignment to or compatibility with existing clinical guidelines for treating obesity in CYP, perceived barriers and facilitators, referral practices, and work infrastructure relating to managing obesity in CYP. CFIR constructs addressing the outer setting, inner setting, characteristics of individuals, and process were embedded in the survey. There were 10 service-oriented factors surveyed based on CFIR constructs. Of these aforementioned factors, CFIR sub-domains from the inner setting identified as relevant to the local health system were as follows: *available resources; structural characteristics* (work infrastructure); *compatibility*; *networks*; *and communications and access to knowledge and information*. The relevant sub-domains used in the survey from the outer setting domain were *patient needs and resources* and *cosmopolitanism.* Further, the sub-domains were *individual stage of change* and *self-efficacy* from the characteristics of individuals domain and *reflecting and evaluating* related to current practices from the implementation process domain. Further details of CFIR constructs used in the survey are provided in Supplementary Appendix 3. In addition, open-ended questions provided participants with an opportunity to highlight ideas and insights related to treating obesity in CYP in Ireland.

## Data analysis

Stata 17 was used for data analysis. Descriptive statistics were used and reported as proportions and means. Categorical variables were compared using chi-square tests. Reported *p*-values are two-tailed and *p* < 0.05 was considered significant. To determine the differences in obesity assessment and treatment in CYP by HPs’ roles, we recategorised HPs’ roles into four groups for analysis as follows: (i) doctors/physicians (general practitioner, area medical officer, paediatrician, self-employed physician/doctor, consultant doctor, non-consultant hospital doctor, surgeon, dentist, and orthodontist), (ii) nurses (general nurse, practice nurse, hospital nurse, and public health nurse), (iii) health and social care professionals (HSCPs) (dietitians, pharmacists, physiotherapists, psychologists, occupational therapists, social workers, and speech and language therapists), and (iv) other professionals (community health workers, public health coordinators, health promotion officers, environmental health officers, clinical managers, family support workers, and primary healthcare coordinators). Further, Spearman correlations with the Bonferroni correction were performed to explore HPs’ self-efficacy in their practice for obesity management in CYP. Free-text comments were reviewed and mapped to the CFIR constructs used in the survey questions as described above.

## Results

A total of 266 participants started the survey, among which 184 completed it. Participants took an average of 20 min to complete the survey, and surveys were considered complete if the participant answered all the demographic questions and 22 mandatory questions relating to managing paediatric obesity (see Supplementary Appendix 1).

### Participant characteristics

**Figure 2:**
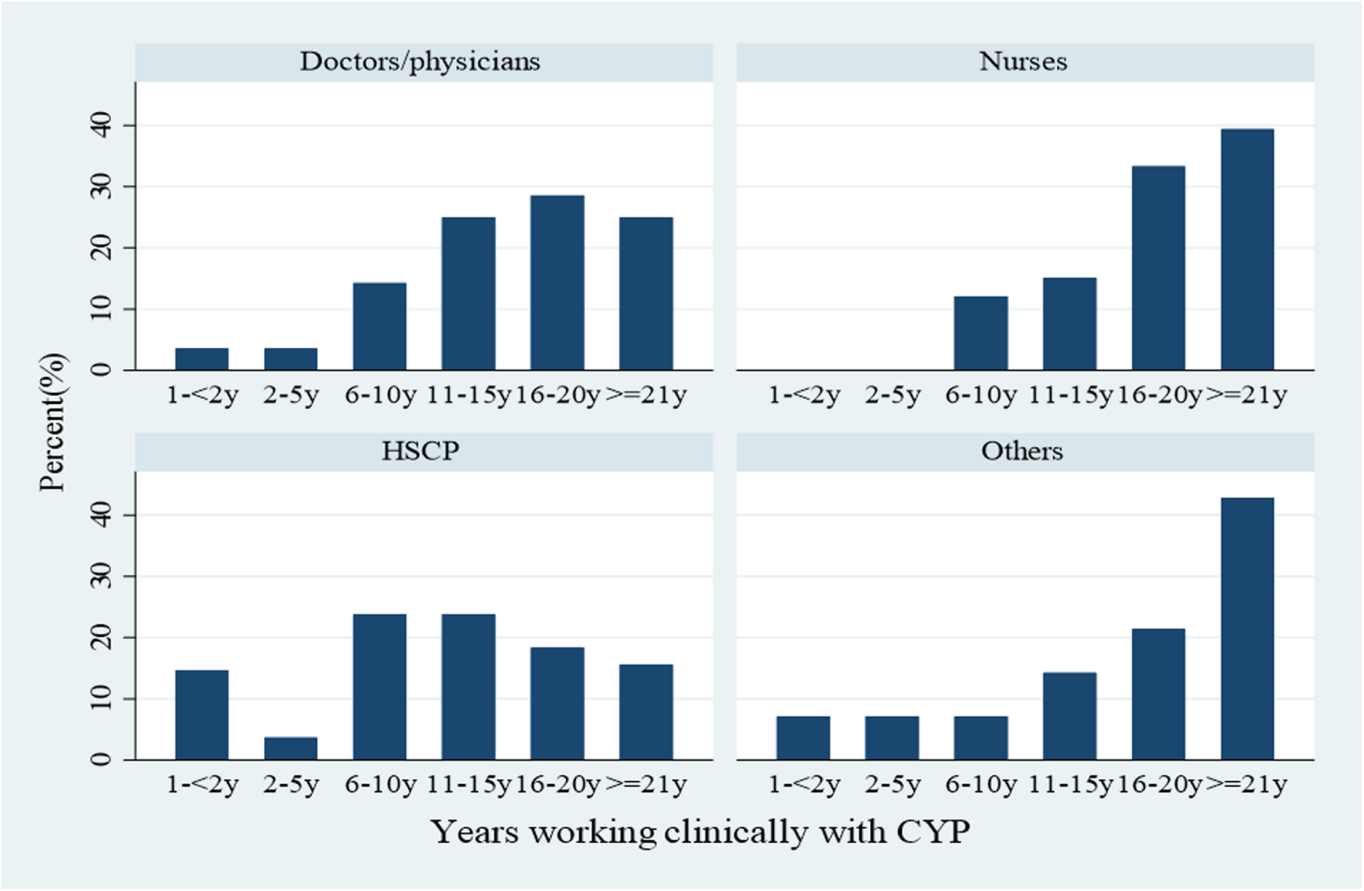
Years working clinically with CYP, *n* = 184.

Of the survey participants, 91% were female, 6% were male, and 3% chose not to specify their gender. Professional diversity was noted among participants with representation from HSCPs (i.e., dietitians, physiotherapists, psychologists, and occupational therapists) nurses, doctors/physicians, and “others” (see Figure 2 and Table 1). Each of the nine community health organisations (CHOs) and seven hospital groups in Ireland were represented, including 24 of the 26 counties in the ROI. Most of the participants (92%) stated their involvement in some components of paediatric obesity care, with 76% involved in obesity care and 40% involved in the care of CYP with severe obesity. Moreover, 3% of the participants reported that they did not provide any care for obesity to those 0–18 years. One-third of the HPs (60/184) reported that they gave an obesity diagnosis to children/adolescents (see Table 1).

**Table 1:** Demographics of healthcare professionals, *N* = 184.

	Frequency (*n*)	Proportion (%)
Gender of HPs		
Female	167	90.8
Male	12	6.5
Prefer not to say	5	2.7
Years of post-graduate/post-registration clinical experience (range, mean ±SD), *n* = 181	1–39	17.9 ± 11.5
<2 years	17	9.2
2–5 years	11	5.9
6–10 years	22	11.9
11–15 years	40	21.7
16–20 years	22	11.9
>21 years	69	37.5
Years working clinically with CYP (range, mean ± SD), *n* = 171	1–42	12.3 ± 8.9
<2 years	29	15.8
2–5 years	15	8.2
6–10 years	35	19.0
11–15 years	27	14.7
16–20 years	33	17.9
>21 years	32	17.4
Clinical role of participants		
Doctors/physicians	26	14.1
Nurses	33	17.9
Health and social care professionals	110	59.8
Others	15	8.2
Clinical site where HPs work^a^		
HSE primary care	87	47.3
Public hospital	50	27.2
Social care/family support/child and adolescent mental health services	17	9.2
General practice	16	8.7
Disability services/rehabilitation hospital	15	8.2
Private practice (health and social care practitioner)	7	3.8
Location of work setting^a^		
City	76	41.3
Suburban	41	22.3
Town	68	36.9
Rural	43	23.4
Not applicable	3	1.6
HP routinely working with CYP who present with^a^		
Underweight	124	67.4
Healthy weight	155	84.0
Overweight	170	92.4
Obesity	140	76.1
Severe obesity	73	39.7
Portion of HP’s clinical role addressing obesity in CYP		
Main role	6	3.3
Part of the general caseload	133	72.3
Neither	45	24.5
Proportion of HP’s caseload working with CYWO, *n* = 178		
0%–10%	79	44.4
20%–30%	69	38.8
40%–50%	21	11.8
60%–70%	5	2.8
80%–90%	–	–
100%	4	2.3
Age of CYP that HPs see routinely in clinical practice^a^		
None of the above	6	3.3
Under 5 years	95	51.6
5–12 years	132	71.7
13–16 years	114	61.9
>16 years	63	34.2
HPs that conduct a clinical assessment in CYP suspected of having obesity as part of their clinical practice	92	50.0
HPs that diagnose obesity in CYP as part of their clinical practice	60	32.6
HPs who accept referrals for treatment of obesity in CYP from those that work with children, *n* = 88	44	50.0

CYP, children and young people.

^a^ Includes multiple responses.

### Evidence of CFIR constructs from the “inner setting domain” related to obesity management for CYP

#### Available resources

One in three HPs (31.7%) reported that they had the time and resources to access training and education in obesity management for CYP, and the response rates varied by HP professional role (3.8% of doctors/physicians vs. 3.3% of nurses vs. 22.4% of HSCPs vs. 2.2% of other professionals; *p* = 0.166). Further, only 31.5% of the HPs reported they had time to undertake a clinical assessment for CYP suspected of having obesity, where responses significantly varied by HP role (4.9% of doctors/physicians vs. 4.4% of nurses vs. 20.7% of HSCPs vs. 1.6% of other professionals; *p* = 0.006) (data not shown in the table).

Nearly half of the HPs reported that they had access to resources used to measure growth in CYP, although this response rate varied significantly by HP role (*p* = 0.007). Only 44% of the HPs reported that they possessed the resources to measure growth in all children/adolescents who needed it, and responses varied by HP role (*p* = 0.007). In addition, 10% of the HPs had either no/limited access to suitable weighing scales and/or height measures or no/limited access to relevant age- and sex-adjusted child growth charts (WHO, UK/Ireland). Only two out of three HPs stated that they had the appropriate measures and tools for undertaking a clinical assessment for CYP suspected of having obesity, and responses varied significantly by HP role (*p* = 0.047) (see Table 2).

**Table 2:** Availability of materials and equipment used for obesity management in CYP, *n* = 184.

	Overall, *n* = 184 (%)	Doctors/physicians, *n* = 26 (%)	Nurses, *n* = 33 (%)	HSCPs, *n* = 110 (%)	Others, *n* = 15 (%)	*p*-value
Availability of tools						
Have the resources needed to measure growth in children/adolescents	90 (48.9)	16 (57.1)	24 (72.7)	43 (39.5)	7 (50.0)	**0**.**007**
Have the resources to measure all children/adolescents that I need to	80 (43.5)	14 (50.0)	21 (63.6)	39 (35.8)	6 (42.9)	**0**.**005**
Prevented from routinely carrying out growth monitoring for children/adolescents						
No/limited access to suitable scales and/or height measures	15 (8.2)	2 (7.1)	2 (6.1)	10 (9.2)	1 (7.1)	0.969
No/limited access to relevant age- and sex-adjusted child growth charts (WHO, United Kingdom/Ireland)	16 (8.7)	3 (10.7)	5 (15.2)	7 (6.4)	1 (7.1)	0.390
Clinical assessment for children/adolescents suspected of having obesity						
Have the measures and tools needed	61 (69.3)	15 (75.0)	14 (87.5)	27 (57.5)	5 (100)	**0**.**047**
Have access to (any) digital/paper tools/resources that need for delivering weight management interventions and treatment, *n* = 86	39 (45.4)	8 (47.1)	3 (21.4)	24 (48.0)	4 (80.0)	0.125
Can offer treatment to all who need it within my caseload, *n* = 85	25 (29.4)	2 (12.5)	2 (13.3)	18 (36.7)	3 (60.0)	0.052

The bold values indicate that there were significant differences within HPs roles for each observation.

#### Structural characteristics (work and information technology infrastructures)

One in five HPs reported having information technology (IT) infrastructure (any IT or digital) to perform a clinical assessment for CYP suspected of having obesity, with responses varying significantly by HP role (6.5% of doctors/physicians vs. 3.8% of nurses vs. 8.2% of HSCPs vs. 1.6% of other professionals; *p* = 0.032) (data not shown in the table).

#### Compatibility

**Figure 3:**
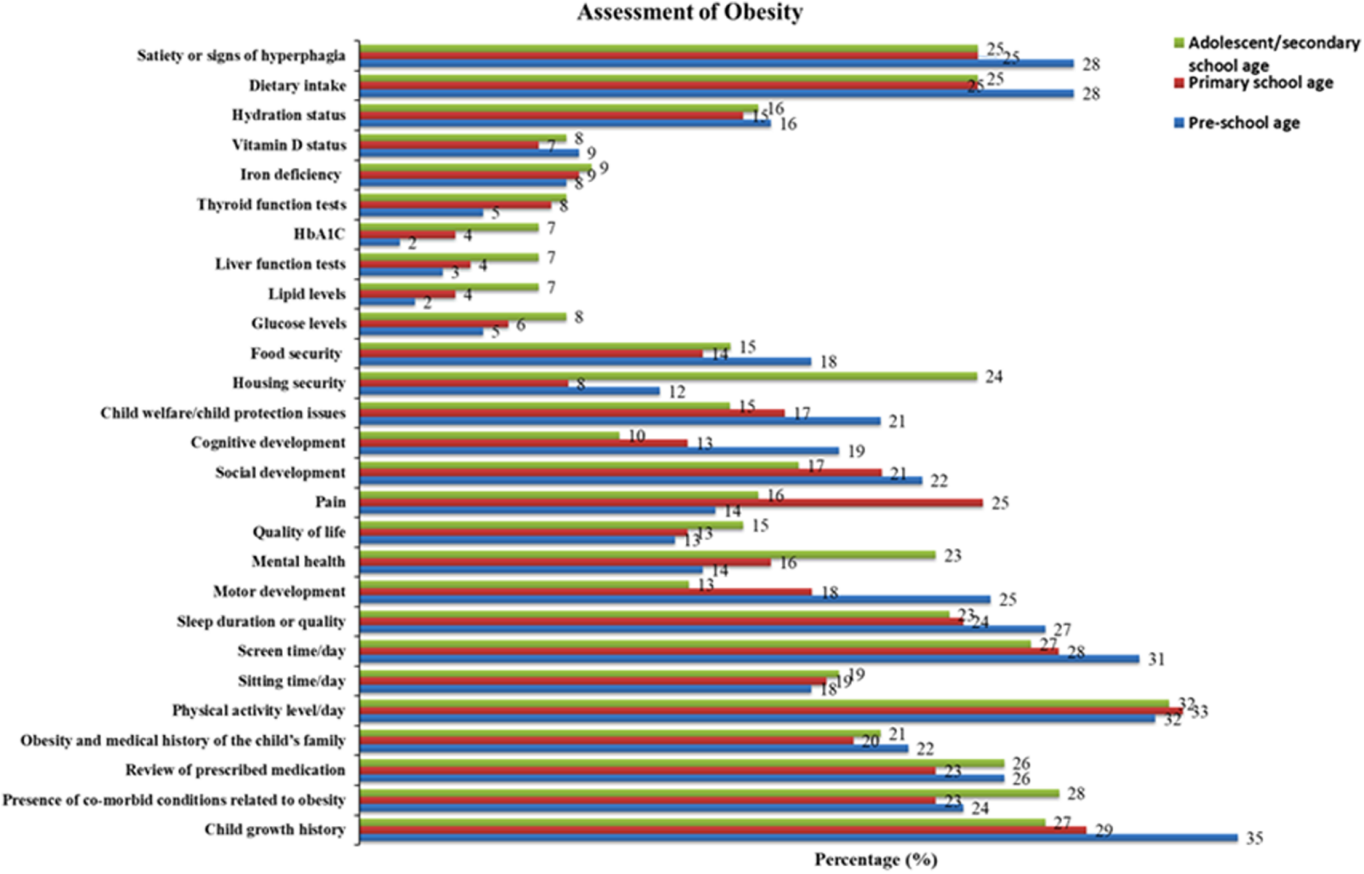
Proportion of HPs who report completing specific components of obesity assessment in CYP of different ages, *n* = 184.

Nearly half of the participants reported either usually or at every appropriate time speaking with parents about child growth measurement, asking permission to measure growth, and conducting growth monitoring in CYP. Nearly 36% of the HPs reported that they routinely followed clinical guidelines or standards for paediatric obesity treatment. HPs reported conducting clinical assessments (any) for obesity management in only 2%–35% of children depending on the age of the child (see Figure 3).

#### Networks and communication

**Figure 4:**
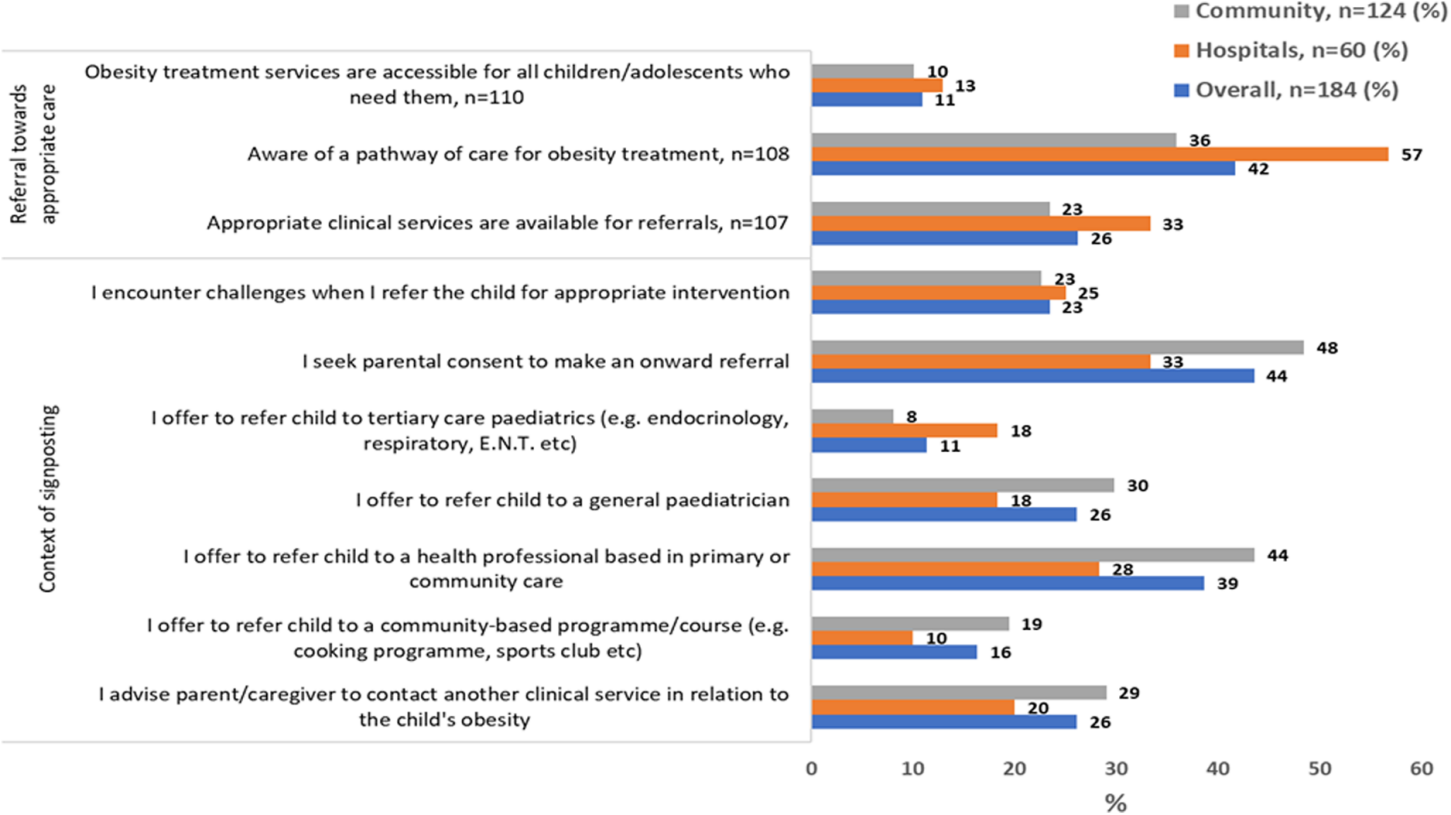
Local conditions, networks, and communications related to paediatric obesity management by clinical setting, *n* = 184.

One in five (*n* = 43/184) participants reported that they seek parental consent to make an onward referral for children/adolescents with obesity (see Figure 4).

#### Access to knowledge and information

Fifteen percent of the HPs reported that they had the expertise to diagnose and stage obesity following a clinical assessment in children and adolescents (Table 3), and 42% of the HPs were aware of a pathway of care for obesity treatment (see Figure 4).

**Table 3:** HP’s self-efficacy related to obesity management in CYP, *n* = 184.

	Overall, *n* = 184 (%)	Doctors/physicians, *n* = 26 (%)	Nurses, *n* = 33 (%)	HSCPs, *n* = 110 (%)	Others, *n* = 15 (%)	*p*-value
HP’s self-efficacy in accessing training and education in weight management for children/adolescents						
I know what training I need	68 (37.4)	8 (28.6)	19 (59.4)	34 (31.5)	7 (50.0)	0.018
HP’s self-efficacy in being adequately skilled in components of obesity management						
Measuring and interpreting growth data	104 (57.1)	20 (74.1)	28 (84.9)	45 (41.7)	11 (78.6)	<0.001
Communicating with parents and their children/adolescent	91 (49.7)	19 (67.9)	23 (69.7)	41 (37.9)	8 (57.1)	0.003
Assessment of obesity-related complications in children	43 (23.6)	10 (37.0)	6 (18.2)	24 (22.2)	3 (21.4)	0.428
Diagnosing obesity and explaining this to parents and their children/adolescents	68 (37.6)	18 (66.7)	10 (30.3)	34 (21.5)	6 (46.2)	0.001
Delivering evidence-based obesity interventions for children	45 (24.6)	6 (21.4)	5 (15.2)	30 (27.5)	4 (30.8)	0.394
Clinical audit or monitoring/evaluating the impact of my treatment	43 (23.8)	10 (37.0)	4 (12.1)	25 (23.4)	4 (28.6)	0.275
Giving an obesity diagnosis and explaining this to parents and their children/adolescents, *n* = 68						
Have the expertise to diagnose and stage obesity following a clinical assessment in children and adolescents	10 (14.7)	1 (5.6)	1 (10.0)	7 (20.6)	1 (16.7)	0.555
Makes an obesity diagnosis for any child/adolescent suspected of having obesity	17 (25.0)	8 (44.4)	—	9 (26.5)	—	0.026
Can explain an obesity diagnosis for any child/adolescent suspected of having obesity using appropriate language that the parent understands	31 (45.6)	9 (50.0)	5 (50.0)	15 (44.1)	2 (33.3)	0.913
Confident in the knowledge of current clinical guidelines for treating obesity in children and adolescents, *n* = 87	21 (24.1)	2 (11.8)	1 (6.7)	15 (30.0)	3 (20.0)	0.043
Professionally and clinically well prepared to manage children with obesity, *n* = 85	27 (31.8)	4 (23.5)	2 (14.3)	17 (34.7)	4 (80.0)	0.055

CYP, children and young people.

### Evidence of CFIR constructs from the “outer setting domain” related to obesity management for CYP

#### Patient needs and resources

Over half of the HPs reported signposting families to local health and/or community services or adult commercial weight management services (see Table 4). HPs reported referral to secondary and tertiary care as described in Figure 4.

**Table 4:** Growth assessment, diagnosis, communication, and signposting by HPs for paediatric obesity management, *n* = 184.

	Overall, *n* = 184 (%)	Doctors/physicians, *n* = 18 (%)	Nurses, *n* = 27 (%)	HSCPs, *n* = 45 (%)	Others, *n* = 7 (%)	*p*-value
Speak with parents about child growth measurement and ask permission for growth measure, *n* = 96						
At every appropriate opportunity	54 (29.4)	4 (4.1)	19 (19.8)	28 (29.2)	3 (3.1)	**0**.**003**
Usually	18 (9.8)	7 (7.3)	—	8 (8.3)	3 (3.1)	
Sometimes	13 (7.1)	4 (4.2)	3 (3.1)	6 (6.3)	—	
Not usually	5 (2.7)	2 (2.1)	1 (1.0)	1 (1.0)	1 (1.0)	
Not at all	6 (3.3)	1 (1.0)	3 (3.1)	2 (2.1)	—	
Not applicable	88 (47.8)	—	—	—	—	
Measure heights and weights in paediatric patients (<18 years), *n* = 96						
At every appropriate opportunity	56 (30.4)	5 (5.2)	18 (18.8)	29 (30.2)	4 (4.2)	0.203
Usually	12 (6.5)	5 (5.2)	1 (1.0)	5 (5.2)	1 (1.0)	
Sometimes	17 (9.2)	5 (5.2)	4 (4.2)	6 (6.3)	2 (2.1)	
Not usually	3 (1.6)	1 (1.0)	—	2 (2.1)	—	
Not at all	8 (4.4)	2 (2.1)	3 (3.1)	3 (3.1)	—	
Not applicable	88 (47.8)	—	—	—	—
Use relevant growth charts (WHO, United Kingdom/Ireland) to plot height and weight, *n* = 97						
At every appropriate opportunity	66 (35.9)	9 (9.3)	20 (20.1)	32 (32.9)	5 (5.2)	0.203
Usually	7 (3.8)	4 (4.1)	—	3 (3.1)	—	
Sometimes	13 (7.1)	2 (2.1)	5 (5.2)	5 (5.2)	1 (1.0)	
Not usually	4 (2.2)	2 (2.1)	–	1 (1.0)	1 (1.0)	
Not at all	7 (3.8)	1 (1.0)	3 (11.5)	4 (4.1)	—	
Not applicable	87 (47.3)	—	—	—	—	
Conduct clinical assessments in children/adolescents suspected of having obesity as part of practice, *n* = 180		*n* = 28	*n* = 32	*n* = 107	*n* = 13	
	92 (51.1)	21 (11.7)	17 (9.4)	49 (27.2)	5 (2.7)	0.033
Provides a diagnosis of obesity for a child/adolescent, *n* = 60		*n* = 28	*n* = 33	*n* = 109	*n* = 14	
Use BMI centiles/SDS alone to diagnose obesity in children and adolescents	39 (21.2)	11 (5.9)	10 (5.4)	15 (8.2)	3 (1.6)	0.011
Use measures of BMI in addition to the presence of complications to diagnose obesity in children and adolescents	25 (13.6)	11 (5.9)	1 (0.5)	12 (6.5)	1 (0.5)	0.001
Delivers obesity services to CYP						
Under 5 years	71 (38.6)	15 (8.2)	13 (7.1)	38 (20.7)	5 (2.7)	0.345
6–12 years	71 (38.6)	14 (7.6)	4 (2.2)	49 (26.6)	4 (2.2)	0.002
13–16 years	64 (34.8)	12 (6.5)	4 (2.2)	45 (24.5)	3 (1.6)	0.007
>16 years	45 (24.5)	9 (4.9)	4 (2.2)	31 (16.9)	1 (0.5)	0.076
None of the above	78 (42.4)	9 (4.9)	14 (7.6)	47 (25.5)	8 (4.4)	0.499
Routinely follows any clinical guidelines or standards for paediatric obesity treatment, *n* = 81	*n* = 81	*n* = 17	*n* = 15	*n* = 44	*n* = 5	
	29 (35.8)	6 (7.4)	6 (7.4)	14 (17.3)	3 (3.7)	0.634
Signposts parent to local health and/or community services for weight management where possible, *n* = 166	*n* = 166	*n* = 28	*n* = 29	*n* = 97	*n* = 12	
	88 (53.0)	16 (9.6)	15 (9.0)	49 (29.5)	8 (4.8)	0.841
Provides signposting to local adult, commercial weight management services, *n* = 166	10 (6.0)	4 (2.4)	2 (1.2)	1 (0.6)	3 (1.8)	0.001

SDS, standard deviation score.

The bold values indicate that there were significant differences within HPs roles for each observation.

#### Cosmopolitanism

Twenty-three percent of the HPs reported that they encounter challenges when they refer a child for appropriate intervention (see Figure 4). Further, we explored agreement with statements regarding experiences and practices for signposting and onward referral for obesity in children/adolescents, among those who responded that they do so. While 41.7% of participants reported they were aware of a pathway of care for the treatment of children/adolescents with obesity, a higher referral rate towards appropriate care for childhood obesity was reported by HPs who worked in hospitals (see Figure 4).

### Evidence of CFIR constructs from the “characteristics of individuals domain” related to obesity management for CYP

#### Individual stage of change

Over half of the HPs (54%) reported conducting growth monitoring in children/adolescents as part of practice, and as such they were classified as “implementation facilitators.” A higher proportion of doctors/physicians (67.9%) and nurses (84.9%) performed growth monitoring in children/adolescents in comparison to that of HSCPs (41.7%) and other professionals (50.0%) as part of practice (*p* < 0.001) (data not shown in the table).

### Evidence of CFIR constructs from the “characteristics of individuals domain” related to obesity management for CYP

#### Self-efficacy

**Figure 5:**
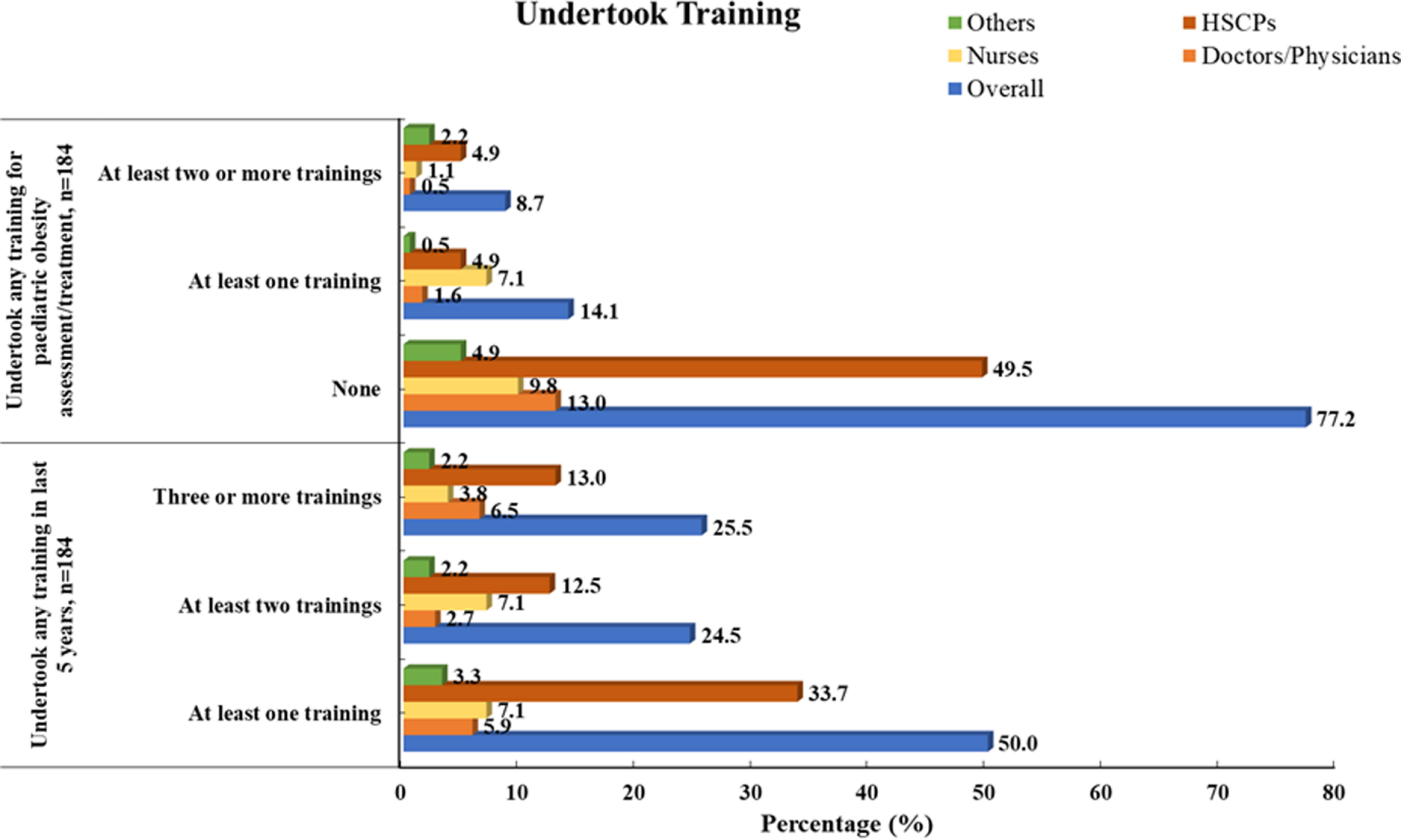
Training received by HPs for obesity management in CYP, *n* = 184.

Almost 60% of the doctors/physicians, 90% of nurses, and 58% of HSCPs worked clinically for over 10 years with CYP (see Figure 1). Overall, 77% (*n* = 142/184) had not received any professional training related to paediatric obesity assessment/treatment, and 8.7% reported completing at least two or more types of training related to paediatric obesity assessment/treatment (see Figure 5). All of the participants (*n* = 184) had confirmed undertaking at least one of the following training courses within the last 5 years; brief interventions training (Making Every Contact Count) (63%), paediatric growth/anthropometric measurements (36%), behaviour change theories/strategies (35%), clinical assessment of paediatric overweight/obesity (27%), referral/signposting to appropriate paediatric obesity management services (23%), approaches to paediatric obesity management (20%), monitoring health outcomes in paediatric obesity (7%), and prescribing medications for paediatric obesity (2%). No professionals reported receiving training in bariatric surgical skills. Although there was variation by HP background, half of the HPs had undertaken at least one type of training course within the previous 5 years, a quarter had undertaken at least two training courses within 5 years, and another quarter had undertaken three or more types of training within 5 years (see Figure 5).

Low levels of self-efficacy regarding the training needs of HPs were reported, whereby 37% reported that they knew what training they needed, and differences were noted between the disciplines (*p* = 0.018) as described in Table 3. There were differences among the HP groups regarding their perception of being adequately skilled in specific components of obesity management and a number of these are to be expected given the professional scope of practice around diagnosis, growth measurement and interpretation and communication with parents and children. Less than 30% of the HPs perceived they had adequate skills in the clinical assessment of obesity-related complications; delivering evidence-based treatment and monitoring/evaluating the impact of treatment (see Table 3). Some of the differences observed between HPs role and CYP obesity management skills. Under half the HPs reported being able to explain an obesity diagnosis using appropriate and understandable language with parents, and under 15% reported having the expertise to diagnose and stage obesity following clinical assessment.

Twenty-four percent of the HPs (*n* = 21/87) were confident in the knowledge of current clinical guidelines for treating obesity in children and adolescents. In addition, 31.8% of the HPs (27/85) perceived they were professionally and clinically well prepared to manage children with obesity (see Table 3).

There were significant positive relationships observed between the perceived ability of HPs to deliver components of child/adolescent obesity management and the type of training received in the past 5 years (see Table 5). There were no statistically significant relationships observed between HPs’ signposting for child/adolescent obesity management and training related to referring/signposting received in the past 5 years. On the other hand, a significant negative association was observed between HP’s perceived awareness of a pathway of care for the treatment of children/adolescents with obesity and training related to referring/signposting received in the past 5 years (*r* = −0.27; *p* < 0.05) (see Supplementary Appendix 4).

**Table 5:** Relationship of the perceived ability of HPs to deliver components of child/adolescent obesity management with the type of training received in the past 5 years, *n* = 184.

Type of training received in the last 5 years	HPs’ perception of adequately trained to					
	Measure and interpret growth data	Communicate with parents and their CYP about growth measurement	Assess obesity-related complications in CYP	Diagnose obesity and explain to parents and their CYP	Deliver evidence-based obesity interventions for CYP	Audit, monitor, or evaluate the impact of treatment
Brief intervention	−0.18*	−0.13	−0.13	−0.08	−0.06	−0.13
Taking growth and anthropometric measures for patients <18 years	0.56*	0.55*	0.20*	0.37*	0.26*	0.02
Clinical assessment of patients <18 years with overweight/obesity	0.40*	0.36*	0.31*	0.35*	0.29*	0.07
Behaviour changes theory and strategies	0.04	0.9	0.14	0.13	0.30*	0.16*
Approaches to weight management for patients <18 years	0.41*	0.37*	0.24*	0.29*	0.31*	0.14
Monitoring health-related outcomes for patients <18 years with overweight/obesity	0.24*	0.24*	0.35*	0.23*	0.29*	0.15*
Referring/signposting families to appropriate weight management services	0.07	0.15*	0.06	0.08	0.07	−0.001
Prescription of medications for children and adolescents with overweight/obesity	0.13	0.08	0.09	0.11	0.09	0.09
Surgical skills training in bariatric medicine	—	—	—	—	—	—

* *p*-value < 0.05.

### Evidence of CFIR constructs from the “process domain” related to obesity management for CYP

#### Reflecting and evaluating

We further asked HPs about the obesity treatment delivered as part of their practice. Two-thirds of the HPs reported providing general obesity prevention advice and education for parents (if indicated), and a quarter of the HPs (41%) reported tailoring brief interventions based on the clinical assessment conducted with the child. Less than 20% of the HPs reported delivery of personalised treatment strategies as recommended in clinical guidelines, less than 15% reported providing opportunities or facilities to develop family skills and behavioural strategies, and half reported monitoring the impact of their treatment (see Table 6).

**Table 6:** HPs’ compatibility to delivery of treatment for paediatric obesity, *n* = 68.

	Overall, *n* (%)
Type of assessment and treatment delivered^a^	
Assessment of the home environment for structures supportive of weight management	15 (22.1)
Assessment of the family’s expectation of weight management	25 (36.8)
Assessment of family’s definitions of a successful outcome in weight management	19 (27.9)
General obesity prevention advice and education for parents if indicated	42 (61.8)
Tailored brief intervention based on the clinical assessment conducted with the child	28 (41.2)
Delivery of a multicomponent behavioural intervention	13 (19.1)
Delivery of family-based group intervention	9 (13.2)
Advice and education in paediatric weight management^a^	
Advice and education regarding dietary intake	44 (64.7)
Advice and education regarding increasing physical activity towards age-appropriate level	43 (63.2)
Advice and education regarding limiting daily screen time	43 (63.2)
Advice and education regarding improving sleep duration and/or quality	37 (54.4)
Advice, education and practice related to behaviour change techniques (e.g., self-monitoring, goal setting)	27 (39.7)
Prescription of appropriate treatment^a^	
Age-appropriate, personalised therapeutic exercise	14 (20.6)
Neuromusculoskeletal/developmental rehabilitation	5 (7.4)
Age-appropriate personalised meal plans/supervised diets	10 (14.7)
Pain management techniques	7 (10.3)
Provision of opportunity and facilities for paediatric weight management^a^	
Provision of opportunities and facilities to engage in supervised physical activity	6 (8.2)
Provision of opportunities and facilities to develop cooking skills	10 (14.7)
Therapeutic counselling to support behaviour change	10 (14.7)
Review the child’s progress^a^	
More than 6 months	30 (44.1)
More than 12 months	20 (29.4)
Monitor the impact of the treatment I deliver	34 (50.0)

^a^ Multiple responses.

### Qualitative evaluation of HPs’ perspectives

At the end of the survey, we sought comments regarding the barriers to ensuring that children/adolescents with obesity have access to healthcare in line with Article 24 of the United Nations (UN) Convention on the Rights of the Child (34). HPs mentioned barriers aligned to the CFIR outer setting domain such as external policy in addition to cosmopolitanism (importance of partnerships and connections). From the inner setting domain, barriers aligned to networks and communications; lower relative priority (deprioritisation of obesity compared to other childhood illnesses and conditions); tension for change (perceived reduced equity for those with obesity in terms of accessing the health service); a lack of access to knowledge, information, and training; and a limited implementation climate to deliver obesity treatment. Barriers related to culture were identified such as limited public awareness and lack of resources for those whom English is not a first language and inaccessible treatment services (see Table 7).

**Table 7:** HPs’ reported barriers in ensuring children/adolescents with obesity can access healthcare in line with the UN Convention on the Rights of the Child.

CFIR constructs’ name			Findings
Main domains	Relative sub-domains	Sub-constructs	
Outer setting domain (the setting in which the Inner Setting exists, e.g., hospital system, school district, state.)	External policy	—	• Some healthcare professionals reported that they do not need training to ensure children/adolescents with obesity can access healthcare in Ireland • Health professional alignment with Health at Every Size principles or intuitive eating models and solely focused on calories and weight • No pathway in place in primary care • Poverty
	Patient needs and resources	—	
Inner setting domain (inner setting regardless of implementation and/or delivery of the innovation, i.e., they are persistent general characteristics of the inner setting)	Network and communication	—	• Availability of information like family-based interventions/community interventions is limited • No specified referral system for childhood obesity • Lack of a structured transitional pathway of care • Lack of clarity in the health service system about the available appropriate services
	Culture		• Parental acceptance of the issue/mindset barriers tackling obesity/unwillingness/drop out for follow-up visits • Unfavourable care service, i.e., current healthcare for paediatric obesity is judgmental and instils shame in the child and family which leads to eating disorders in adulthood • Lack of coordination between the local health services dietitians and disability health services, often refuse to accept patients with obesity • Lack of consideration for this group of children in priority service • The prevention and treatment of overweight and obesity are often a secondary focus
Inner setting domain (implementation and/or delivery of the innovation)	Readiness for implementation	Available resources	• Lack of specialist services outside Dublin • Difficult to access community support services for children/adolescents/families • Distance, i.e., especially for people living in northeast is a big factor in accessing specialised obesity treatment • No/lack of enough public awareness including health staff awareness on appropriate feeding for infants and children • Lack of resources for non-English speaking people/language and ethnicity barrier • Lack of knowledge on available resources/not enough easily readily accessible and accountable services in a timely fashion • Lack of resources in community services
		Access to knowledge and information	• Lack of time/unwillingness of the professionals (i.e., dietitians, GPs) to provide service because of their large caseload • Lack of evidence-based multidisciplinary embedded programmes available to treat obesity • Lack of community dietitian/lack of MDT working in primary care/community • Inadequate evidence-based treatment options • No/lack of training/post-graduate training/limited opportunity to upskill/clinician’s training and confidence (sensitive topic, therefore, can be difficult to discuss with parents) • Lack of understanding of the complexity of obesity management for children • Poor quality of child food • Too much inaccurate information online • Lack of availability of resources, i.e., OECD and DOH 2016–2025 obesity reports do not have open access
	Implementation climate	Relative priority	• Not prioritised, as more urgent referrals exist • Long waiting time (e.g., 2 years–30 months) • Extensive waiting lists and time lag between referral and being seen can hinder addressing the issue
Characteristics of individuals	Self-efficacy		• Low self-efficacy of HPs

MDT, multidisciplinary team.

One of the HPs extended their opinion as follows, “Long waiting lists for Primary Care intervention in many areas. Children with obesity are often not referred to primary care physiotherapy or psychology. Additional barriers [exist] for children attending disability services or CAMHS [child and adolescent health services].”

Another HP elaborated, “Not prioritised as more urgent referrals received. Difficult to make progress individually as often requires a family-based intervention. Parents make initial changes and improvements can be made but often only temporary.”

Furthermore, another HP noted, “Facilitators: Families have been very receptive when they are actually approached. Actual face-to-face training especially on clinical measurements could potentially improve some of the below, as well as training in motivational interview technique. Barriers: There is no pathway in place in primary care, it is so far not a service priority in many services, professionals lack confidence in approaching the topic ‘the difficult conversation’ but also in setting up a service or pathway due to perceived low self-efficacy around the issue.”

## Discussion

The present study observed gaps in guideline understanding and dissimilarity in assessment, counselling, and treatment practices in the ROI for childhood obesity management (29). The study provides rich information on factors considered to be strengths such as a genuine interest from HPs in providing optimal care in addition to multiple actionable barriers identified by HPs. Overall, HPs reported spending time and resources as part of their routine care with CYP in addressing obesity, whereby assessment and growth monitoring were the most reported components of management. HPs identified the need for materials and equipment suitable for all children (including those with disability), adequate training, improved competence and self-esteem, pathways of care, and better equity. According to the HSE model of care, “obesity is a complex, chronic, multifactorial disease that requires a comprehensive multidisciplinary, approach to care across the lifespan” (35). Some HPs commented that they did not consider child/adolescent obesity as a disease which may imply a knowledge gap among care providers and potentially may influence practice related to onward referral, clinical assessment, and signposting to other services. Research from 2015 highlighted divergent clinical opinions between those who consider obesity a disease and those who consider it a risk factor for disease (36), and more recent discussions extend the scientific discussion to encompass situations where obesity can be both a risk factor and a disease in itself (37).

The crucial step in clarifying whether a child has the disease of obesity is the completion of a holistic clinical assessment to identify whether obesity-related complications are present. The initial step of assessing obesity relates to taking anthropometric measures of growth. In the present study, 47% of the HPs reported taking growth measurements in CYP sometimes, usually, or at every opportunity. Differences in this practice existed based on HP background which can be expected (see Table 3). For example, the HSCP group included dietitians who would measure growth as an essential component of their practice. Further, most HPs did not conduct obesity-related clinical assessments other than measuring height, weight, and body mass index (BMI) in school-aged children and adolescents (i.e., growth history, assessment for comorbid conditions, or obesity-related complications). This observation indicates the need for improved clinical training and potentially usable clinical resources like standardised clinical toolkits to optimise the standard of care that CYP with obesity can access. Monitoring obesity-related complications and tailoring treatment to reduce these are essential for arresting the impact of obesity on the developing body and preventing further progression into adolescence and adulthood (29, 36). In this study, HPs reported that they did not have sufficient resources like “time” in their usual practice to conduct clinical assessments (other than growth) for paediatric obesity. These results complement the previous findings that lack of provider knowledge and confidence, lack of time to provide service, limited resources, and lack of access to resources are the perceived barriers by HPs for paediatric obesity management (38–40). Clearly, if the cornerstone of obesity treatment is the provision of age-appropriate education and training in practical skills related to health, nutrition, and supervised physical activity, it is therefore essential to ensure sufficient time for HPs to deliver this in clinical encounters.

The present data showed that HPs’ self-efficacy related to undertaking training on paediatric obesity assessment/treatment was low. Appropriate levels of training for HPs are recommended by the World Health Organization and in Ireland by the Obesity Policy and Action Plan (10, 41) and the Model of Care for the Management of Overweight and Obesity in the ROI (35). In our study, HPs who had undertaken obesity-relevant training in the previous 5 years reported higher levels of perceived ability to deliver components of obesity management. This finding highlights the importance of providing HPs ongoing access to obesity-related training and education. A recent Dutch study advocated empowering HPs with low levels of confidence in obesity management (42). Our study observed that a higher proportion of doctors and nurses reported higher self-efficacy for measuring and interpreting growth data and communicating with parents and their children/adolescent compared to the other HP groups. It is expected that in the “others” group, self-efficacy in this task would be low as the practice of measuring and communicating is likely not part of their daily work. For the HSCP group, some of these HPs (e.g., dietitians) are likely very engaged in the practice of measuring and communicating, whereas other HSCP groups (e.g., physiotherapists or psychologists) may not be. In addition, though HSCPs, doctors and nurses may be engaged in the practice there may be particular factors related to why the level of self-efficacy differed for completing the tasks. For example in other recent work, German researchers investigated whether overconfidence in a healthcare skill was related to motivation to receive training in that skill (43). The authors observed that the three distinct groups of HPs emerged with one “recruitable” group who showed mild overconfidence with some motivation to attend training and an “unaware” group who was highly overconfident in their skill level but incompetent in practice and lacked motivation for training. Observed differences may have been due to the acculturated nature of work and interest/motivation in training that could be threatened by overconfidence (43). In our study, when HPs did see children with overweight/obesity, half of them reported a lack of referral pathways and were not clear what support was available for families. Previous studies across the world revealed that a lack of referral pathways was a deterrent to the provision of child/adolescent weight management (29, 35, 38). Limited resources or limited access to resources such as BMI charts and materials were cited as a further barrier to management in the present study (44), and few HPs had the available information or technology infrastructure to conduct clinical assessments which limits the ability to deliver integrated care services for paediatric obesity (45).

This study has several strengths including the recruitment of a broad range of participants who hailed from a variety of healthcare settings, professional disciplines, and regions and the use of an evidence-based survey to assess the service-oriented gaps, barriers, and management strategies being used in practice. Our survey was co-designed with patient advocates, mixed-methods researchers, and expert clinicians and was guided by a systematic review of clinical practice guidelines for treating obesity in CYP. The co-creation process ensured that issues and processes deemed important by those with lived experience of receiving obesity treatment were included. Additionally, one strength of the study was the employment of the CFIR framework to help categorise data related to the implementation of obesity interventions known to affect the translation of evidence into practice. To our knowledge, our survey is the first to collect data regarding the specific components of evidence-based obesity treatment in CYP from a broad range of HPs involved in delivering healthcare to CYP. We used different methods of recruitment in this study including collaboration with professional bodies, policymakers, and healthcare managers and development of a bespoke social media campaign with engaging digital assets (46). Another strength includes the broad regional coverage of participants who represent the whole of the ROI where there are existing and well-published health inequalities and disparities. The study results are limited by the small number of HPs who undertook the survey. Although this study may not be generalisable for all HPs in the ROI, the reader should keep in mind that Ireland is a small country with a population of just over five million people. The field of paediatrics is underdeveloped in Ireland, and, currently, it is not known how many healthcare professionals work with children and adolescents. As such, it is not possible to calculate what proportion of paediatric healthcare professionals who participated in the survey. In addition, the results may be influenced by selection bias, as HP participation was voluntary and participants were likely those interested in this area of practice rather than reflecting the views and experiences of all HPs working with children and adolescents.

## Conclusions

Our data provide practical information to improve pathways of care and implementation of services for assessing and treating obesity in children and adolescents in the ROI. These findings suggest that HP’s knowledge of appropriate care for paediatric obesity is inconsistent with evidence-based recommendations. To improve professionals’ self-efficacy, HPs must increase access to clinical training and education so that evidence-based treatment can be delivered to maximise health outcomes for children with obesity. The findings of this study will be used in a subsequent qualitative study to explore specific implementation barriers and facilitators and to prioritise them for how they might be feasibly addressed.

## Data Availability

The raw data supporting the conclusions of this article will be made available by the authors, without undue reservation.
