# Supplementary material for: Addressing child and adolescent obesity management in Ireland: identifying facilitators and barriers in clinical practice
Source: Front Pediatr. 2023 Jul 7;11:1222604. doi: 10.3389/fped.2023.1222604 (PMC10365269; doi:10.3389/fped.2023.1222604)
Supplement: Supplementary file 1 [file Datasheet1.pdf]

Appendix-1. Survey

Childhood Obesity LANDSCAPE Study: survey for healthcare professionals

You are being invited to take part in an online research study to be carried out by the Obesity Research and Care Group at RCSI, in collaboration with the HSE. This is an anonymous questionnaire to assess current practice relating to child and adolescent weight management in the Republic of Ireland, and will take between 10-25 minutes, depending on your level of involvement in weight management.

Before you decide whether or not you wish to take part, you should read the information carefully. Please feel free to ask questions about the study before deciding, by contacting the researcher, Louise Tully (louisetully@rcsi.com) or the principal investigator (graceomalley@rcsi.com).

Download the participant information sheet here. This includes all information about the study and your data protection.

Childhood Obesity LANDSCAPE Study: survey for healthcare professionals

Data protection information and consent form

Please select yes or no for the following statements. Please note that if you answer no to any of the below, you cannot proceed with participation and will be redirected to the end of the questionnaire.

* 1. I have read and understood the participant information sheet about this research project. The information has been fully explained to me and I have been able to ask questions by contacting the research team if needed.

○ Yes

○ No

* 2. I understand that I don't have to take part in this study and that I can opt out at any time. I understand that I don't have to give a reason for opting out.

○ Yes

○ No

* 3. I am aware of the potential risks of this research study.

◯ Yes

◯ No

* 4. I have been given the opportunity to download a copy of the participant information sheet and this completed consent form for my records.

◯ Yes

◯ No

* 5. I consent to take part in this research study having been fully informed of the risks.

◯ Yes

◯ No

* 6. I give informed explicit consent to have my data (collected anonymously) processed as part of this research study.

◯ Yes

◯ No

Childhood Obesity LANDSCAPE Study: survey for healthcare professionals

7. Please select one or more of the following, related to your preferences for future use and retention of the anonymous data collected for this survey. <u>You do not need to select yes to any of these options in order to proceed.</u>

	Yes	No
OPTION 1: I give permission for data to be stored for possible future research related to the current study only if consent is obtained at the time of the future research but only if the	◯	◯

research is approved by a Research Ethics Committee.		
OPTION 2: I give permission for data to be stored for possible future research related to the current study without further consent being required but only if the research is approved by a Research Ethics Committee.	◯	◯
OPTION 3: I give permission for data to be stored for possible future research unrelated to the current study only if consent is obtained at the time of the future research but only if the research is approved by a Research Ethics Committee.	◯	◯
OPTION 4: I give permission for data to be stored for possible future research unrelated to the current study without further consent being required but only if the research is approved by a Research Ethics Committee.	◯	◯
OPTION 5: I agree that some future research projects may be carried out by researchers working for commercial/pharmaceutical companies.	◯	◯
OPTION 6: I understand I will not be entitled to a share of any profits that may arise from the future use of my data or products derived from it.	◯	◯

Childhood Obesity LANDSCAPE Study: survey for healthcare professionals

This questionnaire contains items to assess what aspects of paediatric weight management, if any, you currently carry out, for children and adolescents living with obesity in Ireland.
Some participants may have completed a Sláintecare needs assessment survey from our group in 2020, related to the provision of health professional training and education for childhood obesity. This survey follows on from that work, with the aim to develop an understanding of what factors can facilitate or hamper the provision and access to weight management services for children and adolescents with obesity. While some of the survey questions are similar, we are really keen to delve deeper into your experience and insight so that we can improve services together.

For the purposes of this survey we define paediatric weight management services as any consultation with a registered/accredited health or social care professional that addresses any of the following:

· the assessment of growth and/or body composition and the identification of obesity

· the assessment of physical, psychological/emotional, or family/child welfare concerns or complications related to obesity

· treatment/care of obesity and/or related complications through delivery of age-appropriate advice and education, nutritional therapy, exercise therapy, psychological/counselling or family therapy, medical or assistive devices, social care or welfare interventions, medications, or metabolic surgical procedures

Childhood Obesity LANDSCAPE Study: survey for healthcare professionals

Demographics

Please tell us a little about yourself to help us understand your perspective.

* 8. Please indicate which type of clinical setting you work in (tick all that apply)?

- [ ] General Practice
- [ ] HSE Primary Care
- [ ] Social Care Services
- [ ] Family Support
- [ ] Child & Adolescent Mental Health Services
- [ ] Public Hospital
- [ ] Private Hospital/Clinic
- [ ] Private Practice (Health and Social Care Practitioner)
- [ ] Other (please specify)

* 9. Please indicate what county you work in (please tick all that apply).

- [ ] Carlow
- [ ] Cavan
- [ ] Clare
- [ ] Cork
- [ ] Donegal
- [ ] Dublin
- [ ] Galway
- [ ] Kerry
- [ ] Kildare
- [ ] Kilkenny
- [ ] Laois
- [ ] Leitrim

- [ ] Limerick
- [ ] Longford
- [ ] Louth
- [ ] Mayo
- [ ] Meath
- [ ] Monaghan
- [ ] Offaly
- [ ] Roscommon
- [ ] Sligo
- [ ] Tipperary
- [ ] Waterford
- [ ] Westmeath
- [ ] Wexford
- [ ] Wicklow

* 10. If applicable, please indicate which hospital group you work in? (please tick all that apply)

- [ ] Not applicable
- [ ] Children's Health Ireland
- [ ] Dublin Midlands Hospital Group
- [ ] Ireland East Hospital Group
- [ ] Royal College of Surgeons in Ireland (RCSI) Hospital Group
- [ ] Saolta Hospital Group
- [ ] South/Southwest Hospital Group
- [ ] University of Limerick Hospitals

* 11. If applicable, which Community Healthcare Organisation (CHO) do you work in? (please tick all that apply)

- [ ] Not applicable
- [ ] CHO 1 (Donegal, Sligo/Leitrim/West Cavan, Cavan/Monaghan)
- [ ] CHO 2 (Galway, Roscommon, Mayo)
- [ ] CHO 3 (Clare, Limerick, North Tipperary/East Limerick)
- [ ] CHO 4 (Kerry, North Cork, North Lee, South Lee, West Cork)
- [ ] CHO 5 (South Tipperary, Carlow, Kilkenny, Waterford, Wexford)
- [ ] CHO 6 (Wicklow, Dun Laoghaire, Dublin South East)
- [ ] CHO 7 (Kildare/West Wicklow, Dublin West, Dublin South City, Dublin South West)
- [ ] CHO 8 (Laois/Offaly, Longford/Westmeath, Louth/Meath)
- [ ] CHO 9 (Dublin North, Dublin North Central, Dublin North West)

* 12. If applicable, which TUSLA regional group do you work in? (please tick all that apply)

- [ ] Not applicable
- [ ] Carlow, Kilkenny and South Tipperary
- [ ] Cavan and Monaghan
- [ ] Cork
- [ ] Donegal
- [ ] Dublin North City
- [ ] Dublin South West, Kildare and West Wicklow
- [ ] Dublin South East and Wicklow
- [ ] Dublin South Central
- [ ] Galway and Roscommon
- [ ] Kerry
- [ ] Louth and Meath
- [ ] Mayo
- [ ] Midlands
- [ ] Mid West
- [ ] North Dublin
- [ ] Sligo, Leitrim and West Cavan
- [ ] Waterford and Wexford

13. If none of the above apply, please indicate which private practice or other setting you work in.

* 14. Which of the following best describes your practice location? (please tick all that apply)

- [ ] City
- [ ] Suburban
- [ ] Town
- [ ] Rural
- [ ] Not applicable

* 15. Which of the following do you identify your gender as?

- ◯ A man
- ◯ A woman
- ◯ A transman
- ◯ A transwoman
- ◯ Non binary gender
- ◯ Prefer not to say

* 16. Please indicate your role in the healthcare team (please tick all that apply).

- [ ] Clinical manager
- [ ] Consultant (employed by the HSE)
- [ ] Community health worker
- [ ] Dental surgeon
- [ ] Consultant (employed privately)
- [ ] Dentist
- [ ] Dietitian
- [ ] Environmental health officer
- [ ] Family support worker

- [ ] GP
- [ ] GP nurse
- [ ] Health promotion officer
- [ ] Health researcher
- [ ] Mental health coordinator
- [ ] Non consultant hospital doctor (NCHD)
- [ ] Nurse (practice)
- [ ] Nurse (hospital)
- [ ] Occupational therapist
- [ ] Nurse (public health)
- [ ] Orthodontist
- [ ] Pharmacist
- [ ] Physiotherapist
- [ ] Psychiatrist
- [ ] Psychologist
- [ ] Primary healthcare coordinator
- [ ] Self-employed physician/doctor
- [ ] Speech and language therapist
- [ ] Social worker
- [ ] Surgeon
- [ ] Other (please specify)

* 17. Please indicate the number of years of postgraduate/post-registration clinical experience you have?

* 18. Please indicate the number of years you have worked clinically with patients who are under 18 years?

* 19. In your clinical practice, do you routinely work with children and/or adolescents who present with (tick all that apply)

- [ ] Underweight?
- [ ] Healthy weight?
- [ ] Overweight?
- [ ] Obesity?
- [ ] Severe obesity?

* 20. Is working with patients 0-18 years who present with overweight and obesity

- ◯ Your main role?
- ◯ Part of your general caseload?
- ◯ Neither?

Other (please specify)

21. On average, what percentage of your caseload do you estimate are children/adolescents with overweight or obesity?

* 22. From which of the following age groups do you routinely see children/adolescents with overweight or obesity in your practice? (tick all that apply)

- ☐ Under 5 years
- ☐ 5-12 years
- ☐ 13-16 years
- ☐ > 16 years
- ☐ None of the above

Childhood Obesity LANDSCAPE Study: survey for healthcare professionals

Accessing and undertaking training in paediatric weight management

* 23. In the last five years, have you had any training in (tick all that apply):

- ☐ Brief interventions (e.g. Making Every Contact Count)?
- ☐ Taking growth and anthropometric measures for patients <18 years?
- ☐ Clinical assessment of patients <18 years with overweight/obesity?
- ☐ Behaviour change theory and strategies?
- ☐ Approaches to weight management for patients <18 years?
- ☐ Monitoring health related outcomes for patients <18 years with overweight/obesity?
- ☐ Referring/signposting families to appropriate weight management services?
- ☐ Prescription of medications for children and adolescents with overweight/obesity?
- ☐ Surgical skills training in bariatric medicine?

* 24. What type of training for paediatric obesity assessment/treatment have you received/undertaken? (tick more than one box if appropriate)

- ☐ No training received
- ☐ Self-directed study
- ☐ Short Inservice training
- ☐ Day-course
- ☐ CME Small Group Topic
- ☐ Topic Certificate
- ☐ Diploma
- ☐ ICGP Learning Module
- ☐ Sláintecare Childhood Obesity Education Online Course
- ☐ Masters degree
- ☐ Doctoral degree

Other (please specify)

* 25. Related to accessing training and education in weight management for children/adolescents, please indicate whether the following statements are true

	Yes	No
I know what training I need	○	○
I know where to find training I need	○	○
I have the time and resources to access training	○	○

* 26. For which of the following do you feel you are adequately trained (tick all that apply):

	Yes	No	Not applicable to my practice
Measuring and interpreting growth data	◯	◯	◯
Communicating with parents and their children/adolescents about growth measurement	◯	◯	◯
Assessment of obesity-related complications in children/adolescents	◯	◯	◯
Diagnosing obesity and explaining this to parents and their children/adolescents	◯	◯	◯
Delivering evidence-based obesity interventions for children/adolescents	◯	◯	◯
Clinical audit or monitoring/evaluating the impact of my treatment	◯	◯	◯

Childhood Obesity LANDSCAPE Study: survey for healthcare professionals

Measuring general growth and related health outcomes in children and adolescents

* 27. I conduct growth monitoring in children/adolescents as part of my practice

- ☐ Yes
- ☐ No

28. I have the resources I need to measure growth in children/adolescents

- [ ] Yes
- [ ] No
- [ ] Other (please specify)

29. I have the resources to measure all children/adolescents I need to

- ◯ Yes
- ◯ No
- ◯ Other (please specify)

30. Please indicate your current practice related to child growth monitoring, by rating on a scale of 1-5 with one being 'not at all', and five being 'at every appropriate opportunity'.

	1	2	3	4	5
I speak with parents about child growth measurement and ask permission to measure their child	◯	◯	◯	◯	◯
I measure heights, and weights in patients <18 years	◯	◯	◯	◯	◯
I use relevant growth charts (WHO, UK/Ireland) to plot height and weight	◯	◯	◯	◯	◯

31. Do any of the following prevent you from routinely carrying out growth monitoring for children/adolescents (tick all that apply)?

- ☐ No access to suitable scales and/or height measures
- ☐ Limited access to suitable scales and/or height measures
- ☐ No access to relevant age- and sex-adjusted child growth charts (WHO, UK/Ireland)
- ☐ Limited access to relevant age- and sex-adjusted child growth charts (WHO, UK/Ireland
- ☐ Limited/no access to other necessary resources (please specify)

32. Please add additional barriers or details.

Childhood Obesity LANDSCAPE Study: survey for healthcare professionals

Clinical Assessment for children/adolescents suspected of having obesity

This section related to children/adolescents who have a large body size, and for whom you expect may have obesity.

* 33. I conduct clinical assessments in children/adolescents suspected of having obesity as part of my practice

- ◯ Yes
- ◯ No

Childhood Obesity LANDSCAPE Study: survey for healthcare professionals

Clinical Assessment for children/adolescents suspected of having obesity continued

This section related to children/adolescents who have a large body size, and for whom

you expect may have obesity.

34. Please tick any of the following that apply to you in relation to undertaking a clinical assessment for children/ adolescents suspected of having obesity

	Yes	No
I have the time	○	○
I have the measures and tools I need	○	○
I have any IT or digital resources/equipment I need	○	○

35. What health-related factors do you assess for children undergoing a clinical assessment for obesity? Tick all that apply, or leave blank if you do not carry out these tests as part of a weight management assessment.

	Pre-school age	Primary-school age	Adolescent/secondary school age
Child's growth history	☐	☐	☐
Presence of co-morbid conditions related to obesity (asthma, depression, hypertension, sleep apnoea, type 2 diabetes, incontinence, musculoskeletal pain)	☐	☐	☐
Review of prescribed medications	☐	☐	☐
Obesity and medical history of the child's family	☐	☐	☐
Physical activity level per day	☐	☐	☐
Sitting time per day	☐	☐	☐
Screen time per day	☐	☐	☐

Sleep duration or quality	☐	☐	☐
Motor development	☐	☐	☐
Mental health	☐	☐	☐
Quality of life	☐	☐	☐
Pain	☐	☐	☐
Social development	☐	☐	☐
Cognitive development	☐	☐	☐
Child welfare/ child protection issues	☐	☐	☐
Housing security	☐	☐	☐
Food security (whether family have resources to provide regular nutritious meals for children)	☐	☐	☐
Glucose levels	☐	☐	☐
Lipids	☐	☐	☐
Liver function tests	☐	☐	☐
HbA1C	☐	☐	☐
Thyroid function tests	☐	☐	☐
Iron deficiency	☐	☐	☐
Vitamin D Status	☐	☐	☐
Hydration status	☐	☐	☐
Dietary intake	☐	☐	☐
Satiety or signs of hyperphagia	☐	☐	☐

Childhood Obesity LANDSCAPE Study: survey for healthcare professionals

Diagnosing obesity in children who are above a healthy weight

* 36. I diagnose obesity in children and adolescents as part of my clinical practice

◯ Yes

◯ No

Childhood Obesity LANDSCAPE Study: survey for healthcare professionals

Diagnosing obesity in children who are above a healthy weight continued

37. Please tick any of the following that apply to you in relation to making an obesity diagnosis for a child/adolescent

☐ I use BMI centiles/SDS alone to diagnose obesity in children and adolescents

☐ I use measures of BMI in addition to the presence of complications to diagnose obesity in children and adolescents

☐ I have the expertise to diagnose and stage obesity following a clinical assessment in children and adolescents

☐ I can make an obesity diagnosis for any child/adolescent that I suspect as having obesity

☐ I can explain an obesity diagnosis for any child/adolescent that I suspect as having obesity using appropriate language that the parent understands

Childhood Obesity LANDSCAPE Study: survey for healthcare professionals

Treatment delivery for child/adolescent obesity

38. I deliver approaches to clinical treatment/management of obesity and related complications in children/adolescents as part of my practice for the following age groups (this may include one or multiple aspects of weight management)

- [ ] Under 5s
- [ ] 6-12 years
- [ ] 13-16 years
- [ ] > 16 years
- [ ] None of the above

Childhood Obesity LANDSCAPE Study: survey for healthcare professionals

Treatment delivery for child/adolescent obesity continued

39. Do you currently accept referrals for weight management (treatment of obesity and related complications) in children/adolescents?

- ◯ Yes
- ◯ No

Optional additional information

40. What are the criteria needed for you to accept a referral for paediatric weight management based on (tick all that apply)?

☐ Not applicable

☐ Age

☐ BMI

☐ BMI percentile/z-score

☐ Staffing availability

☐ Waiting list times

☐ I do not know

☐ Other (please specify)

41. What is the average waiting time for new referrals for paediatric obesity in your clinical service?

○ Not applicable

○ 0-6 months

○ 6-12 months

○ 12-18 months

○ 18-24 months

○ >24 months

○ I do not know

42. Do you routinely follow any clinical guidelines or standards for paediatric obesity treatment?

○ No

○ Yes (please specify)

[illegible]

43. Please indicate which of the following you provide as part of your practice, within the context of delivering paediatric weight management (tick all that apply)

- [ ] Assessment of the home environment for structures supportive of weight management
- [ ] Assessment of the family’s expectation of weight management
- [ ] Assessment of family’s definitions of a successful outcome in weight management
- [ ] General obesity prevention advice and education for parents if indicated
- [ ] Tailored brief intervention based on clinical assessment conducted with child
- [ ] Delivery of a multicomponent behavioural intervention
- [ ] Delivery of family-based group intervention
- [ ] Advice and education regarding dietary intake
- [ ] Advice and education regarding increasing physical activity towards age-appropriate level
- [ ] Advice and education regarding limiting daily screen time
- [ ] Advice and education regarding improving sleep duration and/or quality
- [ ] Advice, education and practice related to behavior change techniques (e.g. self-monitoring, goal setting)
- [ ] Prescription of age-appropriate, personalised therapeutic exercise
- [ ] Prescription of neuro/musculoskeletal/developmental rehabilitation
- [ ] Prescription of age-appropriate personalised meal plans/supervised diets
- [ ] Prescription of pain management techniques
- [ ] Provision of opportunity and facilities to engage in supervised physical activity
- [ ] Provision of opportunity and facilities to develop cooking skills
- [ ] Therapeutic counselling to support behaviour change
- [ ] Adjustment of prescribed medications based on potential impact on appetite or weight gain
- [ ] Prescription of obesity medication approved for paediatric use
- [ ] Prescription of obesity medication off-label for paediatric use
- [ ] Review the child’s progress for more than six months

☐ Review the child’s progress for more than 12 months

☐ Other (please specify)

44. Please state whether the following statements apply to you:

	Yes	No
I am confident in my knowledge of current clinical guidelines for treating obesity in children and adolescents	○	○
I am professionally and clinically well prepared to manage children with obesity	○	○
I have access any digital/paper tools/resources I need for delivering weight management interventions and treatment	○	○
I monitor the impact of the treatment I deliver	○	○
I can offer treatment to all who need it within my caseload	○	○

Childhood Obesity LANDSCAPE Study: survey for healthcare professionals

Signposting and referring to external weight management services

* 45. For children/adolescents with obesity, I signpost the parent to local health and/or community services for weight management where possible:

◯ Yes

◯ No (this option will skip the next page, which contains additional questions on signposting)

◯ Comment

[ ]

* 46. For my patients who are children/adolescents with obesity, I provide signposting to local adult, commercial weight management services:

◯ No

◯ Yes (please specify)

[ ]

Childhood Obesity LANDSCAPE Study: survey for healthcare professionals

Signposting and referring to external weight management services continued

47. Please indicate which of the following you currently carry out in the context of signposting for children/adolescents with obesity (tick all that apply)

- [ ] I advise the parent/caregiver to contact another clinical service in relation to the child's obesity
- [ ] I offer to refer child to a community-based programme/course (e.g. cooking programme, sports club etc)
- [ ] I offer to refer child to a health professional based in primary or community care
- [ ] I offer to refer child to a general paediatrician
- [ ] I offer to refer child to tertiary care paediatrics (e.g. endocrinology, respiratory, E.N.T. etc)
- [ ] I seek parental consent to make an onward referral
- [ ] I encounter challenges when I refer the child for appropriate intervention

48. Please indicate whether the following statements apply to you regarding care for children/adolescents with obesity.

	Yes	No
Appropriate clinical services are available to which I can refer children/adolescents with obesity	○	○
I am aware of a pathway of care for treatment of children/adolescents with obesity	○	○
Obesity treatment services are accessible for all children/adolescents who need them	○	○

Childhood Obesity LANDSCAPE Study: survey for healthcare professionals

Anything else?

49. The UN convention on the rights of the child states the right of every child to healthcare (Article 24). If you would like to add any more information about the barriers or facilitators you have encountered in ensuring children/adolescents with obesity can access healthcare in Ireland, please feel free to add elaborate in the comment box below.

Childhood Obesity LANDSCAPE Study: survey for healthcare professionals

Thank you and focus group expression of interest

Thank you very much for providing your time to help us understand the current service landscape for paediatric weight management in Ireland. This will help to form an invaluable basis for further exploring the barriers and enablers to offering best practice care for the next part of this study.

If you would be willing to further discuss barriers, enablers, or supports needed for your current clinical practice in relation to child and adolescent weight management (including any aspect of care in which you may be involved such as referrals, measurements, assessment, diagnosis, treatment and monitoring), please click this link so we can securely capture your contact details on a separate platform in order to keep this questionnaire anonymous. If you decide to provide your contact details, they will be stored securely and only used to invite you to take part in a focus group interview and prioritisation workshop later in the year, which you are welcome to ignore or decline once you receive this invite.

Appendix 2: Digital Recruitment Fliers and Posts as Part of Social Media Recruitment Campaign

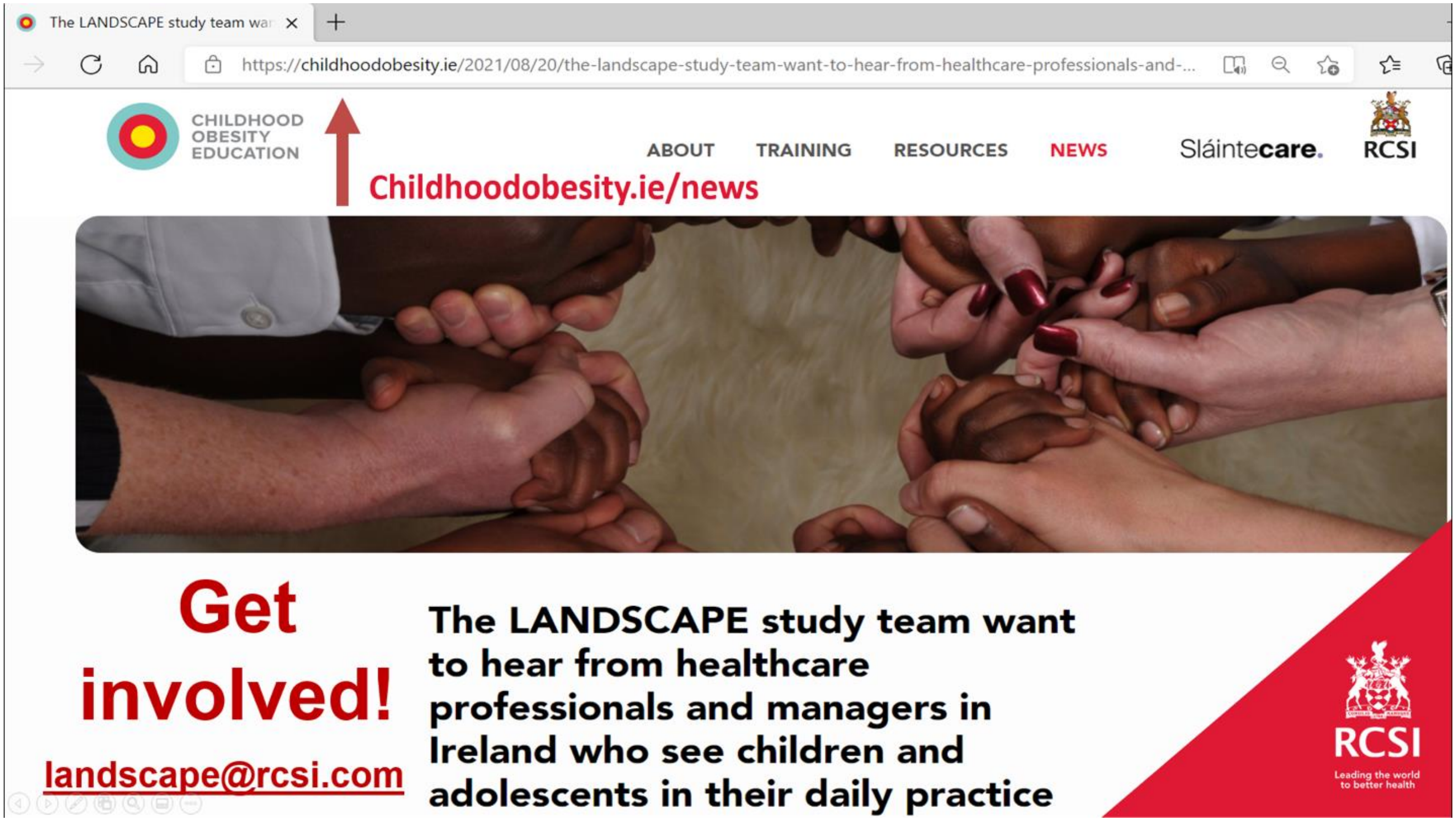

Final call for

Health & Social Care Professionals in Ireland

Do you see children in your practice?

- Nurses
- HSCPs
- GPs/physicians
- Psychologists
- Pharmacists

Help us to document the priority needs for childhood obesity management in practice, or have your say on the topic

Appendix 3: CFIR Constructs addressed in this study.

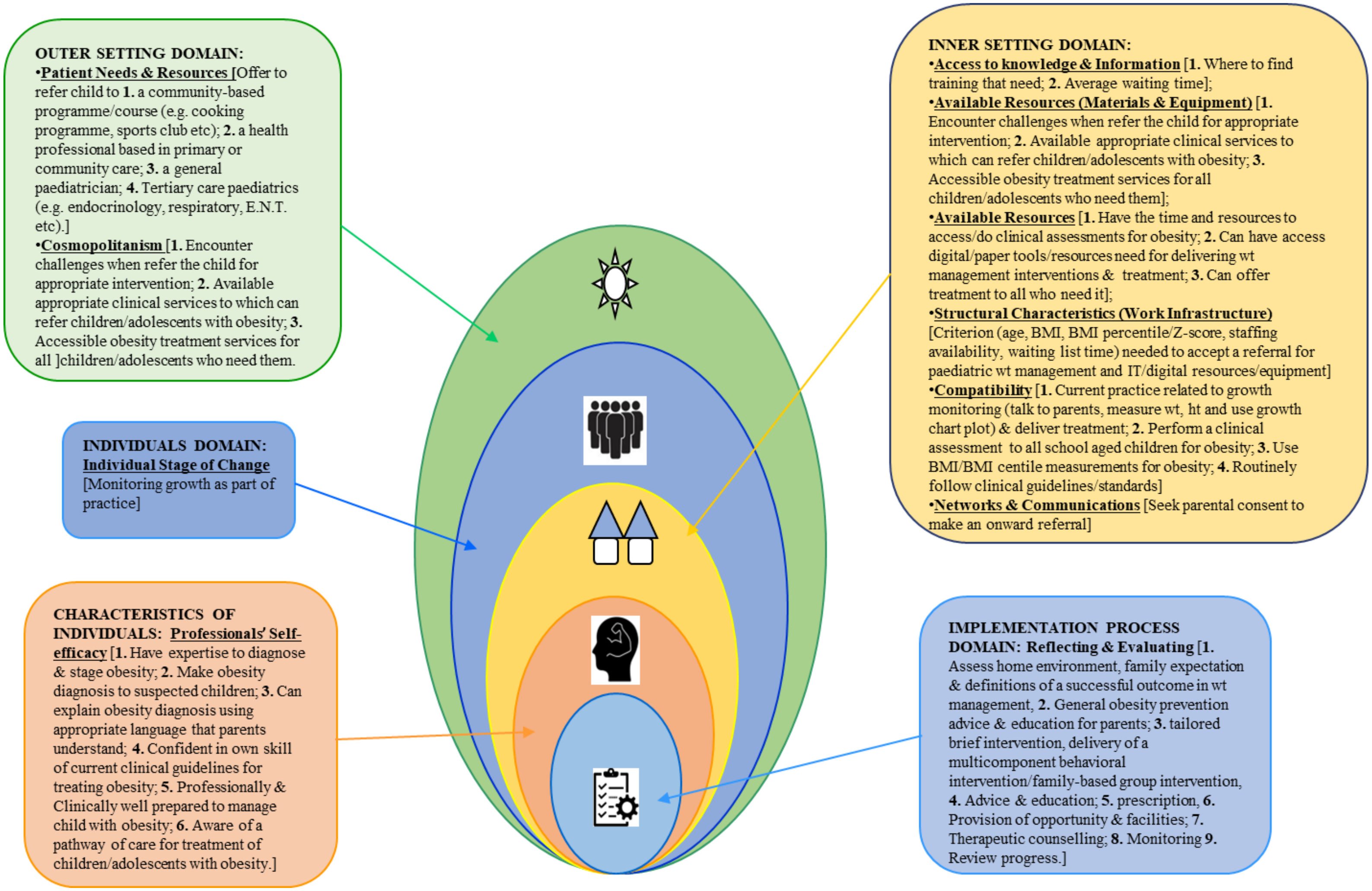

Appendix 4. Relationship between referral/signposting for obesity treatment and type of training received in past 5 years, n=184

		Context of sign posting							Referral toward appropriate care		
		I advise parent/ caregiver to contact another clinical service in relation to the child's obesity	I offer to refer to a community-based programme/ course	I offer to refer child to a HP based in primary or community care	I offer to refer child to a general paediatrician	I offer to refer child to tertiary care paediatrics (e.g., endocrinology, respiratory, E.N.T. etc.)	I seek parental consent to make an onward referral	I encounter challenges when I refer the child for appropriate intervention	Appropriate clinical services are available to which I can refer children/ adolescents with obesity	I am aware of a pathway of care for treatment of children/ adolescents with obesity	Obesity treatment services are accessible for all children/ adolescents who need them
**Type of training received in last 5 years**	Referring/ signposting families to appropriate weight management services	0.14	0.17	0.12	0.17	0.20	0.16	0.18	-0.004	-0.27*	-0.005

*p-value<0.05
